# Insight into Virulence and Mechanisms of Amphotericin B Resistance in the *Candida haemulonii* Complex

**DOI:** 10.3390/jof10090615

**Published:** 2024-08-28

**Authors:** Yuyan Huang, Yanyu Su, Xinfei Chen, Meng Xiao, Yingchun Xu

**Affiliations:** 1 Department of Laboratory Medicine, State Key Laboratory of Complex Severe and Rare Diseases, Peking Union Medical College Hospital, Chinese Academy of Medical Science and Peking Union Medical College, Beijing 100730, China; b2023001071@pumc.edu.cn (Y.H.); estey010023@163.com (Y.S.); chenxinfei29@163.com (X.C.); 2 Beijing Key Laboratory for Mechanisms Research and Precision Diagnosis of Invasive Fungal Diseases (BZ0447), Beijing 100730, China; 3 Graduate School, Chinese Academy of Medical Science and Peking Union Medical College, Beijing 100730, China

**Keywords:** *Candida haemulonii* complex, virulence, antifungal resistance, amphotericin B

## Abstract

The *Candida haemulonii* complex includes emerging opportunistic human fungal pathogens with documented multidrug-resistance profiles. It comprises *Candida haemulonii sensu stricto*, *Candida haemulonii* var. *vulnera*, *Candida duobushaemulonii*, *Candida pseudohaemulonii*, and *Candida vulturna*. In recent years, rates of clinical isolation of strains from this complex have increased in multiple countries, including China, Malaysia, and Brazil. Biofilm formation, hydrolytic enzymes, surface interaction properties, phenotype switching and cell aggregation abilities, extracellular vesicles production, stress response, and immune evasion help these fungi to infect the host and exert pathological effects. Multidrug resistance profiles also enhance the threat they pose; they exhibit low susceptibility to echinocandins and azoles and an intrinsic resistance to amphotericin B (AMB), the first fungal-specific antibiotic. AMB is commonly employed in antifungal treatments, and it acts via several known mechanisms. Given the propensity of clinical *Candida* species to initiate bloodstream infections, clarifying how *C. haemulonii* resists AMB is of critical clinical importance. This review outlines our present understanding of the *C. haemulonii* complex’s virulence factors, the mechanisms of action of AMB, and the mechanisms underlying AMB resistance.

## 1. Introduction

Infections caused by fungi of the *Candida* genus have emerged as a significant medical risk and are characterized by elevated morbidity and mortality rates. The phenomena of globalization and climate change marked by global warming, as well as the growth of immunocompromised populations, have led to the emergence of uncommon and novel species such as *Candida* pathogens in recent decades [1]. The primary source of *Candida* infections and the most extensively researched species is *Candida albicans.* However, there is a rising pattern of infections from non-*albicans Candida* (NAC) species [2], such as *C. tropicalis*, *C. krusei*, *C. parapsilosis*, *C. glabrata*, *C. auris*, *C. lusitaniae*, and the *C. haemulonii* complex.

The species composition of the *C. haemulonii* complex is currently controversial. The mainstream view classifies *C. haemulonii sensu stricto*, *C. haemulonii* var. *vulnera*, *C. pseudohaemulonii*, *C. vulturna*, and *C. duobushaemulonii* species as constituent members of the complex. Initially identified in 1962 as *Torulopsis haemulonii*, *C. haemulonii* was first extracted from the digestive tract of a fish (*Haemulon sciurus*) [3]. The initial clinical isolation of the strain, from a patient’s blood, occurred in 1984 [4]. Lehmann et al. have outlined two separate genetic categories (group I and group II, specifically *C. haemulonii sensu stricto* and *C. duobushaemulonii*) and *C. haemulonii* var. *vulnera*; these categories are distinguished by isoenzyme profiles, DNA relatedness, and physiological traits [5]. Sugita et al. introduced *C. pseudohaemulonii* as a new addition to this complex in 2006 [6]. In 2016, a new related species, later named *C. vulturna*, was isolated from flowers; this species has since been isolated from multiple types of clinical samples [7].

In recent decades, the rates of clinical isolation of strains from this complex have increased in multiple countries, including Malaysia [8], China [9,10], Brazil, and the United States [11]. Particularly in Brazil, the rate of occurrence of the *C. haemulonii* complex rose from 0.9% to 1.7% over the past decade [12]. In China, according to data reported by CHIF-NET, epidemiological tracing of the *C. haemulonii* complex has become more challenging [13]. Until recently, the *C. haemulonii* complex was often either incorrectly identified or overlooked. In addition, closely related strains have often been mistaken for members of the complex. The most commonly misidentified species in this regard is the close relative, *C. auris* [14]. These factors have hindered the generation of data regarding the occurrence and epidemiological patterns of this complex.

This complex’s components are highly infective, especially in the context of patients with weakened immune systems or those suffering from other severe illnesses [15]. Unlike *C. auris*, *C. haemulonii* was previously thought to lack the ability to cause outbreaks in healthcare settings [16]. However, recent studies have identified hospital-acquired outbreaks linked to the *C. haemulonii* complex, including a *C. vulturna* outbreak among individuals in Shanxi Province, China [7]. Similarly, a Panamanian hospital recorded the transmission of both *C. haemulonii sensu stricto* and *C. duobushaemulonii* within its premises [17]. The rate of isolation of *C. haemulonii* clones has also increased, corresponding with considerable resistance to antifungal treatments and suggesting elevated transmission rates [10]. An additional notable aspect of this complex is its propensity to cause breakouts among newborns and older adults [16].

Like other *Candida* species, *C. haemulonii* is known to induce both superficial and systemic infections [10,17]. Clinical infections with different strains of this complex have different symptomatologic features. For example, systemic candidiasis triggered by *C. haemulonii sensu stricto* is characterized by onychomycosis, peritonitis, leukocytosis, and high fever and is linked to peripheral vascular disease, ulcers in the lower limbs, and diabetes mellitus [18,19,20]. *C. duobushaemulonii* is responsible for inducing onychomycosis and vulvovaginal candidiasis, and this species is notably common among patients with diabetes [21,22]. *C. haemulonii* var. *vulnera* is known to lead to multiple conditions, including onychomycosis, ulcers in the lower limbs, and fungemia [23,24].

The majority of *C. haemulonii sensu stricto* and *C. pseudohaemulonii* cases stem from bloodstream infections and occur in individuals with central venous catheters [25]. In fact, the insertion of a central venous catheter and extended durations in intensive care units are known to be prominent risks for the onset of a systemic infection [18], though *C. haemulonii* complex spp. have been isolated from a variety of clinical sites [26]. Interestingly, strains originating from diverse clinical backgrounds exhibit distinct physiological characteristics. For example, Ramos et al. discovered that fungal isolates from body fluids exhibited notably higher proliferation rates (planktonic growth) compared to those from cutaneous candidiasis. At the same time, the viability of biofilms was notably greater in cutaneous isolates than from body fluids [27]. As strains from the *C. haemulonii* complex can cause a high proportion of bloodstream infections [11,28,29], and because relatively little is known about virulence mechanisms, more attention should be paid to the virulence profiles of these species [30].

The complex is notably characterized by its inherent resistance to amphotericin B (AMB), its resistance or minimal sensitivity to azoles [19], and its resistance or limited susceptibility to echinocandins [31]. Efflux pump activity has been shown to be significantly higher in the *C. haemulonii* complex than in the other non-*albicans Candida* species (e.g., *C. tropicalis*, *C. krusei*, and *C. lusitaniae*) [32], which accounts for its minimal vulnerability to azoles. The potential existence of resistance to echinocandin may be linked to mutations in the *FSK* gene [31], as has been demonstrated in *C. parapsilosis* [33]. AMB targets ergosterol, and its mechanisms of action are mainly related to pore formation, polyene–sterol interactions, ergosterol sequestration, oxidative stress, and metabolic status, but the extent to which each of these mechanisms plays a role remains controversial [34,35]. While it is known that AMB primarily acts as an external drug, the mechanisms of resistance to AMB remain particularly unclear. This review focuses on the virulence factors of the *C. haemulonii* complex, the mechanisms of action of AMB, and factors leading to antifungal resistance, with particular focus on clinical applications.

## 2. Virulence Factors

Species from the *C. haemulonii* complex tend to infect immunocompromised patients and cause outbreaks in healthcare settings. This invasiveness can be attributed to the primary virulence factors that characterize many species in this genus. These factors include the capability to generate extracellular enzymes, phenotype variability, the ability to interact with multiple surfaces, and biofilm formation during candidemia (Table 1).

### 2.1. Hydrolytic Enzymes

Fungi are well known to possess the ability to break down environmental polymeric compounds into smaller pieces, which are readily assimilated by the cells for use as carbon and nitrogen sources [41]. This trait is evident in the elevated activity of hydrolytic enzymes present in cell extracts and supernatants from conditioned cultures of numerous microorganisms. In *Candida* spp., the capacity of peptidases to break down extracellular matrix (ECM) proteins underlies their function in dismantling host barriers during infection, which both supplies nutrients needed for growth and promotes the infiltration and colonization of host tissue. *Candida* spp. also break down the soluble protein’s hemoglobin (the primary protein in red blood cells) and albumin (a prevalent carrier protein in serum), which has been linked to the absorption of nutrients, including iron and amino acids, in mammalian infections [42,43]. Peptidases from *Candida* spp. have also been implicated in immune evasion due to their ability to digest IgG, a key antibody that mediates humoral defense strategies [44,45].

Souto et al. reported that serine-type peptidases, both secreted from and associated with cells in the *C. haemulonii* complex, can cleave a variety of important host proteins, a critical factor in fungal invasiveness [46]. Ramos et al. identified multiple virulence-related molecules secreted from *C. haemulonii* complex isolates, with all isolates producing aspartic proteases, hemolysins, and siderophores, and the activities of phospholipase, esterase, phytase, and caseinase were also detected [47]. In a related study, the complex was shown to synthesize various virulence-related enzymes, and the activity of esterase was notably enriched in samples obtained from skin candidiasis relative to those from bodily fluids [48]. *C. haemulonii* isolated from skin lesions of Brazilian patients was found to generate proteases and phospholipases, which play direct roles in these fungal infections [49].

### 2.2. Extracellular Vesicles

Extracellular vesicles (EVs) are lipid-bilayer enclosed structures that are released by all organisms [50]. Fungal EVs transport a variety of active biological molecules, such as proteins, nucleic acids, lipids, pigments, toxins, gene regulators, and virulence factors [51,52,53]. Consequently, they are viewed as a mechanism for mediating intercellular communication, which is key to the formation and development of microbial communities during interactions between hosts and pathogens [54,55]. Additionally, fungal EVs modulate the activation of host innate immune responses [56]. Numerous studies have shown that fungal EVs are capable of triggering immune reactions in effector cells like macrophages and neutrophils, influencing phagocytosis, altering macrophage orientation towards M1 or M2, elevating chemokine and cytokine concentrations, and boosting the production of reactive oxygen species (ROS) [57,58,59].

Oliveira et al. demonstrated that low levels of *C. haemulonii* EVs can elude detection by macrophages and avoid destruction by the traditional oxidative burst route produced by macrophages, potentially offering a benefit in facilitating the movement of virulence factors via EVs; these EVs that remain undetected by the host immune system potentially serve as precise tube regulators during infection [36]. Conversely, elevated EV concentrations produced by *C. haemulonii* var. *vulnera* were found to activate the microbicidal effect in macrophages [36]. These studies indicated that EVs contribute to the virulence of *C. haemulonii* and serve as potential targets for future treatments.

### 2.3. Phenotypic Switching and Filamentation

The various forms of *Candida* species, as well as their adaptability, are factors that allow them to adjust to constantly evolving environments, particularly in the context of infection. The yeast-filament transition and the white-opaque switch in the extensively researched *C. albicans* are prime illustrations of a phenotypic switching mechanism [60,61]. These processes have been correlated with mating, aspartyl protease (SAP) secretion, and virulence [62,63,64]. In *C. auris*, Deng et al. used glycerol to induce mutations in the *GFC1* gene, which encodes a transcription factor. These mutations triggered a rod-like filamentation-competent phenotype that causes a higher skin fungal burden [65]. Fan et al. identified five cellular morphologies adopted by *C. auris*, of which the filamentous cell type is the most virulent, suggesting a strong connection between form and virulence [66]. In a drug-resistant *C. auris*, Fan et al. also observed a higher rate of the filamentous form and lower copies of the Zorro3 retrotransposon as compared with susceptible isolate; however, whether these correlations are due to causative links between morphology and virulence remains unclear [67].

*C. haemulonii*, a close relative of *C. auris*, was found by Deng et al. to form two new cell types, pink and filamentous, and this discovery was accompanied by the identification of two heritable phenotypic switching systems: a primary white-pink switch and a secondary yeast-filament switch. Historically, it had been believed that *C. haemulonii* lacks the ability for phenotypic alteration or filamentous development, but recent work has suggested that clinical isolates might possess the capability to alternate between these two phenotypes and that environmental elements can influence the morphological changes. Furthermore, an RNA sequencing study revealed differential expression of genes connected with virulence in white and pink cells, encompassing those related to carbohydrate metabolism and morphology, as well as genes encoding transcription regulators involved in filamentous regulation. Consistent with these findings, the three phenotypes were found to exhibit distinct virulence levels, with white cells in *C. haemulonii* being the most virulent, followed by pink cells, and finally filamentous cells [37].

### 2.4. Cell Aggregation

Recent focus has been placed on an aggregative phenotype in *C. haemulonii* due to its primary relationship with anomalies in cell division and its potential role in immune evasion. The aggregative phenotype is suggested to result from an impairment in cell division and an inability to disperse daughter cells following the budding process [68]. The relationship between the aggregation phenotype and the virulence of pathogens remains controversial, and it stems from earlier studies on *C. auris*. Specifically, Borman et al. demonstrated that in a *Galleria mellonella* larvae model, *C. auris* isolates colonizing in aggregate exhibited lower virulence than did their non-aggregative counterparts, and interestingly, these aggregative isolates developed more robust biofilms [68]. Conversely, the work of Bing et al. showed that strains that have mutated to become more aggregative exhibit higher virulence compared to wild type strains, and they found that the mutated genes in evolved aggregative strains play roles in controlling cell division, cytoskeletal properties, and cellular polarity [69]. Interestingly, Carvajal et al. failed to detect a definitive relationship between aggregation phenotype and the virulence of *C. auris* strains via direct infection of *G. mellonella* larvae [70].

Ramos et al. revealed that aggregation typically varies over time, with most of the examined isolates of *C. haemulonii* complex species showing significant aggregation following a 2-h incubation at 37 °C, while the fungal cells that formed these aggregations stayed alive [48]. Ramos et al. also identified a positive correlation between the cell aggregation morphology of *C. haemulonii* and cell surface hydrophobicity (CSH) [71]. CSH has been implicated in the virulence of multiple *Candida* species, including *C. albicans* and *C. tropicalis*, where it contributes to the promotion of biofilm formation, adherence, and the regulation of lipid metabolism [72,73,74]. However, additional research is required to more clearly understand the molecular elements of this fascinating phenomenon.

### 2.5. Surface Interaction Properties

The properties with which pathogens interact with various surfaces have important impacts on virulence. As has been extensively documented, pathogenic *Candida* spp. exhibit diverse virulence characteristics and infection progression due to differences in adherence to inactive and active surfaces, the formation of biofilms, the ability to penetrate host cells and tissues, and the ability to evade innate and adaptive immune defenses [75,76]. From this perspective, the secure attachment of *Candida* spp. and other fungal pathogens to various non-living and living surfaces marks a crucial phase in colonization and the initiation of infections [38,77]. Therefore, surface interaction properties can be considered key aspects of virulence.

Molecules on the surfaces of fungal cells facilitate the early stages of interactions between fungi and host structures. These molecules include various glycoconjugates, such as polysaccharides, glycoproteins, and glycolipids, that are essential for attaching to host elements. Adherence to host tissues and medical devices, such as central or urinary catheters, can lead to biofilm formation and challenging infections. The hydrophobic cells of *C. albicans* show greater adherence to various host tissues compared to their hydrophilic equivalents, and they possess stronger resistance to phagocytic destruction [78]. Beyond CSH and surface charge, specific microbial surface components, including adhesin-like molecules, also play a role in adhesion capacity [79,80].

Ramos et al. conducted a series of studies to assess the effects of fungal cell surface characteristics on the ability to aggregate, the ability to adhere to non-living and living surfaces, and the in vivo aggressiveness of 12 Brazilian clinical isolates of *C. haemulonii* complex in a *G. mellonella* larval model [71]. Electron microscopy showed that round yeasts with uneven surfaces containing protrusions were best able to aggregate. Ultrastructural characterization indicated the presence of amorphous material in these aggregations, with properties similar to that of the ECM. In vitro, *C. haemulonii* complex yeasts with a negative surface charge showed greater adherence to surfaces with a positive charge, and the clinical isolates exhibited interactions with phagocytic cells that correlated with the quantity of mannose- and glucose-rich molecules. These findings demonstrated the capacity of the *C. haemulonii* complex to generate potential virulence traits related to surfaces and that this capacity was a crucial element involved in the mechanisms of infection [71].

### 2.6. Biofilm Formation

Biofilm formation has long been recognized for its contributions to virulence in bacterial and fungal infections, and it is also likely to be directly associated with drug resistance. It is widely held that biofilm formation is a favored structural pattern for many microorganisms. These biofilms are marked by an intricate network of microorganisms engaging with one another and a biotic/abiotic exterior enveloped by a self-produced ECM primarily made up of proteins, polysaccharides, lipids, nucleic acids, minerals, and water [81]. In terms of functionality, the ECM is crucial for the formation, maintenance, and structure of biofilms, offering a defense against external stress factors like immune reactions of the host and challenges with chemicals, including disinfectants or antimicrobial substances; accordingly, biofilms have a direct effect on treatments, particularly in critically ill patients [82]. The survival of biofilms has been shown to be related to the source of the strain, and it is notably greater in skin isolates compared to isolates from bodily fluids [27].

Ramos et al. found that all isolates from the *C. haemulonii* complex are capable of forming biofilms on a variety of inert surfaces, such as urinary, nasoenteric, and nasogastric catheters, which are formed from siliconized latex, polyurethane, and polyvinyl chloride, respectively [26]. Microscopic images revealed that the biofilm structure is densely packed with yeasts accompanied by water channels and an ECM. The primary constituents of the ECM were found to be proteins and carbohydrates, with smaller amounts of nucleic acids and sterols.

Biofilm development is an undeniably important virulence characteristic linked to device-associated candidemia in hospitalized patients [26], and multiple studies have employed animal models to investigate the negative impacts of biofilms. Silva et al. conducted comparative research on several *Candida* species using *G. mellonella* larvae and found that *C. haemulonii* demonstrated a remarkable capacity for biofilm formation in vivo [83]. Lima et al. revealed that the substantial biofilm generation activity of *C. haemulonii* var. *vulnera* and *C. duobushaemulonii* isolates could influence the pathogenic effects in *Caenorhabditis elegans* [84].

### 2.7. Stress Responses

Stress responses enable fungi to survive in the presence of various stresses, including heat shock, oxidative stress, and drug induction; these responses thus contribute to the ability of fungi to infect hosts. *C. auris*, a close relative of *C. haemulonii*, can grow at temperatures of 42 °C [85]. Genomic analyses indicated that the genome of *C. haemulonii* strains includes direct homologs of heat resistance genes, such as those encoding heat shock proteins Hsp60, Hsp70, Hsp90, and Hsp104 [35]. Hsp90 is a highly conserved molecular chaperone that governs temperature-dependent hyphal formation. In *Trichosporon asahii*, Hsp90 has also been associated with resistance to AMB [86]. Additionally, according to research by Kim et al., Hsp90 plays a key role in the morphogenesis, virulence, and azole drug tolerance of *C. auris* [87]. The specific roles of genes encoding heat shock proteins in the pathogenicity of *C. haemulonii* remains to be investigated.

### 2.8. Immune Evasion

*Candida* species have been shown to possess characteristics that permit immune evasion, and the underlying mechanisms are complex. Munoz et al. revealed that the most aggressive species of *C. haemulonii* complex exhibited a marked reduction in phagocytosis, although the hemocyte density remained constant, and no host cell lysis was observed [88]. In *C. albicans*, a trio of genes, *HGT1*, *MSB2*, and *PRA1*, have been shown to play roles in evading immune detection, and potential orthologs of these genes have been identified in *C. haemulonii* complex spp., suggesting that these genes might represent a category of virulence factors [35]. These findings also suggest a resemblance between the methods of immune avoidance in the *C. haemulonii* complex and those employed by *C. albicans*. However, additional research is needed to validate these hypotheses and reveal precise processes.

## 3. Mechanisms of Action of Amphotericin B

AMB, a member of the polyene family, was the first antifungal agent available on the market, and it remains frequently used in antifungal therapy. A key mechanism underlying the polyene’s antifungal action involves binding to ergosterol. Ergosterol plays essential roles in the life processes of fungi, including regulation of membrane proteins, endocytosis, cell division, membrane fluidity, and cell signaling [89]. The key consequences of the interactions of AMB with ergosterol are related to its ability to form pores in fungal lipid bilayers and polar interactions with phospholipid groups in the cell membrane, as well as its ability to sequester ergosterol.

The fact that AMB exists both as a monomer and a polymer is important to its interactions with fungi. When AMB exists as large aggregates, it tends to kill yeasts by extracting ergosterol from membranes, but when the monomeric form predominates, it tends to insert spontaneously into ergosterol-containing membranes to form aqueous pores [90]. The formation of pores leads to the penetration of small ions and electrolytes, which results in an imbalance of intracellular ionic homeostasis [91]. Several studies have also supported a model in which AMB activates intracellular ROS generation, which has a stronger fungicidal effect than the interaction with ergosterol [34,92,93].

### 3.1. Polyene–Ergosterol Interactions

Ergosterol molecules in the fungal cell membrane are a key target of polyenes, including AMB, and polyene–ergosterol interactions are well known to cause damage to fungal cells. However, the mechanisms of interaction remain unclear. Umegawa et al. demonstrated that AMB and ergosterol interact head-to-tail in hydrated phospholipid bilayers [94].

The biological effects caused by the interaction continue to be uncovered. Gottlieb et al. proposed that polyenes could prevent sterol synthesis or competitively replace sterols as a cofactor in an important metabolic reaction [95]. Later studies showed that through the effect on ergosterol molecules, AMB can cause alterations in membrane permeability [96]. These findings have provided insights into the interactions between AMB and sterol, but further confirming studies are needed.

### 3.2. Pore Formation

Pore formation may occur upon the interaction between polyenes and ergosterol, as the resulting complex has been proposed to resemble an ion channel and thus to allow ions and small organic molecules to escape from the cell, ultimately causing cell death [97]. In this model, the hydrophobic tail of the amphipathic polyene interacts with ergosterol and is oriented within the membrane towards the hydrophobic portion of the bilayer, while the hydrophilic part of the polyol forms an aqueous channel [98]. Intermolecular hydrogen bonds between amino and carboxyl groups of the hydrophilic head groups of neighboring polyene molecules further stabilize this channel [97]. Neutron diffraction studies have confirmed that such an architecture can exist [98].

Typically, from 4 to 12 polyene monomers form a pore. As the length of the AMB roughly corresponds to the length of the average membrane phospholipid, two types of channels can be formed: full pores, which are made up of a complex of two polyene rings, and half pores, containing only a single polyene ring. Both types have essentially the same structure, but the half pore induces thinning of the lipid bilayer [97].

The type of polyene and the composition and thickness of the membrane determine the type of pore that is primarily formed [97]. Pore formation occurs only after a threshold level of polyene molecules in the membrane is reached. Below this threshold, aggregated complexes, known as non-aqueous pores or cation-selective pores, increase membrane permeability to monovalent cations. On the other hand, full pores or half pores also allow larger non-electrolyte molecules to pass through. Notably, ergosterol-containing membranes have a substantially lower threshold than do cholesterol-containing membranes [99], in part explaining the specificity of AMB for fungal, as opposed to mammalian, host cells. In addition, patch-clamp experiments have shown that artificial AMB channels in ergosterol-containing membranes are faster ion transporters than those in cholesterol-containing membranes.

The pore diameter determines the selectivity of efflux from the cell. The pore diameter depends on the type and concentration of the polyene antifungal, while the type and concentration of sterols have a much less significant influence. This effect was confirmed by a study by Yang et al., in which the channel diameter was found to increase 100-fold when the concentration of AMB increased by a factor of 40 in an ergosterol-rich membrane [100]. Typically, AMB forms pores of a relatively large diameter, approximately 0.46 nm, thereby facilitating the transport of larger molecules, including sucrose. In comparison, another polyene antifungal agent, nystatin, forms pores of a smaller diameter, approximately 0.36 nm [98].

### 3.3. Surface Adsorption and Sterol Sponging Activity

The second and third modes of polyene action are based on the principle that adsorption or removal of ergosterol destabilizes phospholipid membranes and disrupts essential cellular processes such as endocytosis and regulation of membrane protein function [98]. The surface adsorption model suggests that polyenes can adsorb ergosterol molecules to sequester them to the surface of the phospholipid bilayer. Based on this theory, Anderson et al. conducted a series of investigations into the location and structure of AMB–ergosterol complexes and found that these complexes typically did not fuse at the membrane, but instead formed extramembrane complexes. They hypothesized that by this mechanism, large aggregates of AMB molecules arranged in parallel may form in the membrane, acting as a sterol sponge. Removal of ergosterol from these membranes disrupts a variety of ergosterol-dependent cellular processes, many of which are controlled by membrane proteins that bind directly to ergosterol. This may also explain why resistance to polyene compounds rarely occurs, as alternative membrane sterols, such as lanosterol (an ergosterol precursor), are unlikely to function properly in these processes in drug-resistant cells, thereby reducing cell survival and pathogenicity. They also concluded that the removal of cholesterol from the large extramembrane aggregates of AMB is one of the main causes of its toxicity to mammalian cells, and that optimizing the binding affinity of AMB derivatives to ergosterol could therefore significantly improve their activity [101].

However, this mode of action is controversial. It has been shown that in a cholesterol-saturated environment, such as the mammalian cell membrane, the thermodynamic balance between ergosterol–AMB and cholesterol–AMB would be shifted in favor of the latter; that is, the “sterol sponge” would be saturated with cholesterol instead of ergosterol [98]. Notably, fungal cells also contain a rigid hydrophilic cell wall made of chitin polymers, which can prevent the penetration of hydrophobic ergosterol and thus inhibit the formation of super-aggregates or sponges outside of the cell wall [102].

### 3.4. Oxidative Damage

ROS are natural byproducts of the mitochondrial electron transport chain system. However, excessive accumulation of ROS can cause damage to cellular macromolecules, including the peroxidation of lipids, carboxylation of proteins, and degradation of nucleic acids [103]. Such oxidative damage is widely recognized as a mechanism of antifungal action. For example, during treatment of *C. albicans*, hypoxia, exogenous catalase (CAT), and superoxide dismutase (SOD) mitigate the action of AMB without inhibiting the AMB-induced K^+^ leak current [104]. Similarly, the action of AMB on *C. albicans* cells within biofilms has been shown to correlate with a 50- to 10,000-fold increase in endogenous ROS levels [105]. Several other studies have demonstrated that polyenes, including AMB, induce oxidative stress, leading to DNA damage, protein carbonylation, and lipid peroxidation, ultimately causing or contributing to fungal cell death [106].

### 3.5. Metabolic Alterations

Metabolomic analyses have revealed changes in metabolic status upon treatment of fungi with AMB. For example, in *C. albicans*, it was found that AMB-induced cell death was alleviated by an increase in the production of polyamines (e.g., putrescine, spermidine, and spermine) [107]. Meanwhile, gene expression analysis of *C. albicans* exposed to AMB showed an increase in the expression of stress-related genes in addition to those involved in membrane sterol homeostasis [108]. In *Cryptococcus neoformans*, it was shown that the cells became metabolically inactive after the addition of AMB, and a strong oxidative burst was observed, suggesting that AMB causes cell death in addition to membrane interactions and pore formation [109]. It is not known exactly how this oxidative stress occurs, but it is thought that the binding of polyenes to membranes triggers this reaction and leads to an apoptotic phenotype that generates ROS or that the fungicide itself causes oxidative stress through autooxidation of AMB and generation of free radicals. In the second model, oxidative stress affects polyenes in a way that is distinct from their membrane permeability, although the free radicals generated affect the membrane itself through lipid peroxidation [108].

### 3.6. Formation of Ion Channels

An ion channel model has been proposed to explain the action of AMB on fungi. The research of Dong et al. supported the ion channel model in the context of 16 fungal strains. They found that AMB is highly ordered within the cell membrane and is oriented mainly parallel to the phospholipid acyl chains [110]. AMB has also been found to form ion-permeable channels in cell membranes through interactions with ergosterol molecules [111]. In this process, AMB accumulates in the membranes of ergosterol-containing fungal cells, forming an oligomeric structure that acts as an ion channel [112]. These channels may be responsible for increasing intracellular Ca^2+^ concentrations [99]. Further research into AMB-containing ion channels may assist the development of AMB treatments.

## 4. Mechanisms Leading to Amphotericin B Resistance

Worryingly, studies have shown a high and increasing rate of resistance of *C. haemulonii* isolates to antifungal drugs, especially AMB. Unlike azoles and echinocandins, AMB acts extracellularly, which may lead to some unique resistance mechanisms. Much of the current knowledge about resistance of the *C. haemulonii* complex to AMB involves biofilm formation, membrane composition, membrane permeability, oxidative stress, and metabolic status (Table 2).

### 4.1. Alterations in the Membrane Sterol Composition

As ergosterol, the main sterol in the fungal cell membrane, is a key target of AMB, the proportion of ergosterol and the membrane sterol composition underlie differences in susceptibility. Mutations in some genes that encode proteins in the ergosterol biosynthesis pathway (i.e., *ERG1*, *ERG2*, *ERG3*, *ERG4*, *ERG6*, and *ERG11*) can cause reductions in ergosterol content. This decrease leads to lower susceptibility to AMB at the price of reduced tolerance to external stresses such as high temperatures, oxidative stress, and attack by neutrophils [117]. Silva et al. revealed that the composition of the cell membrane of resistant *C. haemulonii* complex spp. showed a decreased proportion of ergosterol and an increased proportion of ergosterol pathway intermediates, which meant a lower level of the therapeutic target. Treatment with AMB has also been shown to cause a membrane sterol rearrangement that helps the fungal cell to better cope with the environmental stress [91].

Different combinations of antifungal drugs are often used clinically to treat fungal infections due to considerations of drug efficiency, drug safety, and the emergence of clinically multidrug-resistant strains. Kelly et al. reported the cross-resistance of azoles and polyenes [113], and Vazquez et al. showed that pre-exposure to azoles decreased the susceptibilities of *Candida* spp. to AMB by standardized in vitro susceptibility studies [114]. Azoles inhibit lanosterol 14-α-sterol demethylase in the ergosterol biosynthetic pathway, leading to the accumulation of toxic sterols in the membrane and consequently to the alteration of membrane function [96]. A side effect of this azole resistance mechanism is that an absence of ergosterol causes cross-resistance to AMB, whose target is the ergosterol [108,114]. This connection may explain the causative relationship between the two. This phenomenon suggests that treatment of patients with certain antifungal combinations may not be clinically beneficial.

### 4.2. Membrane Permeability

One of the main mechanisms of action of AMB is the formation of pores following its binding to ergosterol in the fungal cell membrane. Therefore, cell membrane permeability may underlie differences of AMB efficacy among various fungal isolates. Pezzotti et al. reported that the action of AMB leads to increased membrane permeability of *C. auris*, resulting in the death of the strain, while resistance to AMB counteracts this effect [118]. Shivarathri et al., using RNA sequencing and measurement of membrane permeability, found that the membrane permeability of clinical susceptible isolates of *C. auris* is 10- to 30-fold higher than that of resistant isolates [115]. These findings collectively suggest that changes in membrane lipid permeability are a critical factor affecting AMB efficacy.

In *C. haemulonii* complex spp., Silva et al. showed that the levels of membrane permeability of resistant isolates are lower than those of susceptible isolates using propidium iodide staining; however, this study showed that high concentrations of AMB could induce only a slight increase in the membrane permeability of *C. haemulonii* complex spp., an increment insufficient to cause cell death [91]. Consequently, the extent to which this property contributes to AMB resistance requires further investigation, considering that AMB’s action is extracellular and involves binding to ergosterol on the fungal cell membrane.

### 4.3. Alterations in the Accumulation of Reactive Oxidative Species

Excessive accumulation of ROS can cause damage in cellular macromolecules such as lipid peroxidation, protein carbonylation, and DNA degradation [103]. Liu et al. found increases in endogenous ROS levels in *C. auris* leads to mitochondrial dysfunction, cytochrome C release, metacaspase activation, and nuclear fragmentation, ultimately resulting in cell death [119]. Accordingly, resistance to ROS-related stress is an important factor that interferes with the killing of *C. auris* by neutrophils [120]. However, the degree of accumulation of ROS in fungal cells can vary from species to species and from isolate to isolate, potentially due to differences in activity of the enzymes responsible for ROS detoxification. In clinical *Aspergillus terreus* isolates, Jukic et al. found that AMB-resistant isolates express higher levels of SOD and CAT and are thus more resistant to oxidative stress, a factor that could be counteracted by the inhibition of SOD and CAT [121].

Linares et al. also found increased levels of SOD and CAT in fluconazole–AMB cross-resistant strains of *C. albicans* and *C. dubliniensis*, and differences between the species were observed, where *C. albicans* expressed significantly higher levels of SOD and CAT compared to *C. dubliniensis* [122]. Consequently, ROS and its detoxification are closely linked to AMB resistance.

Silva et al. revealed that *C. haemulonii* tolerated high concentrations of agents involved in oxidative burst as compared to other non-*albicans Candida* species. The activities of ROS antioxidant enzymes are significantly higher in *C. haemulonii* spp. compared with other non-*albicans* species (*C. tropicalis*, *C. krusei*, and *C. lusitaniae*). After treatment with AMB, *C. haemulonii* isolates did not show a significantly higher level of lipid peroxidation [91]. These studies provide evidence that *C. haemulonii* has a stronger ability to cope with the oxidative stress brought by AMB treatment.

### 4.4. Altered Metabolic Status

Altered metabolic status may play a critical role in AMB resistance. Silva et al. showed that *C. haemulonii* spp. relied more on sugar fermentation rather than aerobic respiration, as evidenced by determination of O_2_ consumption rates, carbon source preferences, and the effects of inhibitors against mitochondrial complexes and alternative oxidases. This shift from aerobic respiration to fermentation may lead to a reduction in the degree of intracellular ROS elevation induced by AMB in *C. haemulonii* [91], thereby increasing the resistance of this strain to AMB.

Future studies are needed to further elucidate the precise mechanisms by which *C. haemulonii* complex spp. cope with oxidative stress and reduce intracellular levels of ROS. Such mechanisms may include enhanced enzymatic ROS scavenging and shifts in respiration patterns.

### 4.5. Altered Iron Homoeostasis

A recent study by Chen et al. found that in *C. albicans*, changes in intracellular iron ion concentrations can affect the membrane sterol composition and membrane permeability [123]. In *C. auris*, Schatzman et al. also found that copper-only superoxide dismutases are induced under iron starvation or during the transition to the filamentous form, thereby reducing the intracellular content of ROS, which may be related to drug resistance [116]. Given the homology of related proteins among *Candida* species, it is possible that *C. haemulonii* complex spp. also experience similar effects of disrupted iron homeostasis, changes in membrane sterol components, increased membrane permeability, and decreased ROS levels. Both changes in membrane sterol composition and permeability, as well as lowering of intracellular ROS levels, could lead to resistance to AMB. Therefore, the relationship between intracellular iron homeostasis and resistance to AMB warrants further investigation.

## 5. Conclusions

The *C. haemulonii* complex is an opportunistic pathogen that readily infects immunocompromised individuals, as well as neonates and older adults. Over the past few decades, there have been increases in the rates of clinical isolation of this complex in several countries, including Malaysia, China, Brazil, and the United States, with alarming outbreaks reported in China. Given the close phylogenetic relationship between *C. haemulonii* and *C. auris*, along with the discovery of certain homologous virulence genes within the *C. haemulonii* complex, there is a potential for high virulence of this species, and further research is warranted to elucidate its virulence profile. In addition, the rate of drug resistance observed for this complex is becoming increasingly alarming, in particular with regard to documented resistance to AMB. While drug resistance mechanisms remain unclear, research suggests that resistance may be associated with the metabolic state of the fungi, oxidative stress, and the composition of the cell membrane.

## Figures and Tables

**Table 1:** Virulence factors and their associated genes.

Virulence Factors	Feature	Possible Related Genes *	Reference
Hydrolytic Enzymes	The capacity of hydrolytic enzymes to break down extracellular matrix proteins allows them to dismantle host barriers during infection, supplying growth nutrients and promoting infiltration and colonization of host tissues.	*SAP*, *LIP5*, *LIP 6*, *LIP7*, *LIP8*, *SAP1*, *SAP2*, *SAP1 SAP3*, *SAP4*, *SAP5*, *PLB1*, *PLB2*, *PLB3*	[35]
Extracellular vesicles (EVs)	EVs facilitate cell-to-cell communication, which mediates interactions between host and pathogen. They modulate the activation of innate immune responses, such as triggering immune reactions in effector cells like macrophages and neutrophils, influencing phagocytosis, altering macrophage orientation towards M1 or M2, elevating chemokine and cytokine concentrations, and boosting reactive oxygen species production.	*NOX2*	[36]
Phenotypic switching and filamentation	Different cell types exhibit different reactions to high temperatures, patterns of gene expression, tolerances to CuSO_4_, activities of secreted aspartyl proteases, and levels of virulence. Consequently, shifts among the three known phenotypes (white, pink, and filament cells) might allow C. haemulonii to swiftly adjust, endure, and prosper in specific host environments, thus enhancing its virulence.	*UME6*, *ACO2*, *KGD2*, *IDP2*, *MDH1-1*, *HGT7*, *HGT17*, *HGT18*, *HGT19*, *CDC11*, *HGC1*, *AGA1*, *SAP3*, *SAP9*, *OFI1*, *PHO84*, *PHO89*, *TSA1*, *GZF3*, *IDH2*, *CPH1*, *HGC1*, *NRG1*, *TUP1*	[35,37]
Cell aggregation	Aggregation phenotypes associated with abnormal cell division may play a role in helping fungi evade host immune defenses.	No genes	
Surface interaction properties	The capacity to stick to both non-living and living surfaces influences exposure in clinical settings (e.g., via medical devices) and propensity of cells to infiltrate host tissues.	*ALS1*, *ALS5*, *ECM33*, *IFF4*, *INT1*, *MP65*, *MNT1*	[35,38,39]
Biofilm formation	Biofilms are characterized by an intricate network among microorganisms, with the biotic/non-biotic exterior being encapsulated by a self-generated extracellular matrix, which consists mainly of proteins, polysaccharides, lipids, nucleic acids, minerals, and water. Biofilms protect against external stressors, such as host immune responses and drugs, and also enhance the virulence of Candida spp., including C. haemulonii.	*IFF4*, *BCR1*, *BRG1*, *EFG1*, *HSP90*, *NDT80*, *ROB1*, *CSR1*	[35,40]
Stress responses	The genomes of *C. haemulonii* strains include direct homologs of heat resistance genes, such as those encoding heat shock proteins Hsp60, Hsp70, Hsp90, and Hsp104. HSP90 plays a key role in the morphogenesis, virulence, and azole drug tolerance of *C. auris*. The specific role of genes encoding heat shock proteins in the pathogenicity of *C. haemulonii* remains to be investigated.	*HSP60*, *HSP104*, *SSA1/HSP70*, *HSP90*	[35]
Immune evasion	C. haemulonii complex can employ a variety of strategies to avoid immune responses, including the creation of biofilms, synthesis of proteases, alteration of morphology, and synthesis of some specific proteins, as well as a number of other undiscovered mechanisms.	*HGT1*, *MSB2*, *PRA1*	[35]

* Note: Potentially related genes were identified solely by sequence homology with genes from *C. albicans* and other well-characterized species, without additional research validation.

**Table 2:** Potential mechanisms of resistance to Amphotericin B.

Mechanisms	Feature	Reference
Alterations in the membrane sterol composition	The biosynthesis of ergosterol, the main sterol of the fungal cell membrane, can be disrupted by genetic mutations, thus leading to decreased susceptibility to amphotericin B (AMB) at the price of increased tolerance of external stresses, such as high temperatures, oxidative stress, and neutrophil attack. Pre-exposure to azoles can inhibit lanosterol 14-α-sterol demethylase, decreasing the production of the AMB target ergosterol. This process results in the build-up of harmful sterols within the membrane and alters membrane function.	[96,108,113,114]
Membrane permeability	Pore formation following interaction with ergosterol is a key mechanism underlying AMB activity. Accordingly, differences in cell membrane permeability can influence AMB efficacy in different fungal isolates.	[115]
Alterations in the accumulation of reactive oxidative species (ROS)	*C. haemulonii* can tolerate high concentrations of oxidative agents compared to other non-*albicans Candida* species, which may correlate with its elevated activity of ROS detoxification enzymes.	[106]
Altered metabolic status	*C. haemulonii* species rely more on sugar fermentation rather than aerobic respiration, thereby decreasing the degree of intracellular ROS elevation.	[91]
Altered iron homoeostasis	In *Candida* spp. that are similar to *C. haemulonii* complex spp., disrupted iron homeostasis leads to changes in membrane sterol composition, increased membrane permeability, and decreased ROS levels that may influence AMB resistance.	[116]
